# Liver Involvement in Children with Hemolytic Uremic Syndrome: Clinical Significance and Prognostic Value

**DOI:** 10.3390/children12121640

**Published:** 2025-12-02

**Authors:** Ezgi Kıran Taşcı, Bora Kunay, Eylem Tazegül Çokgezer, Ezgi Yavuz, Doğan Barut, Pınar Yazıcı Özkaya, Kübra Cebeci, Sevgin Taner, İpek Kaplan Bulut, Miray Karakoyun

**Affiliations:** 1 Pediatric Gastroenterology, Hepatology and Nutrition, Ege University, İzmir 35100, Turkey; ezgikiran@gmail.com (E.K.T.); borakunay@gmail.com (B.K.); eylemtazegul@gmail.com (E.T.Ç.); ogzezgi@gmail.com (E.Y.); barutd047@gmail.com (D.B.); miraykarakoyun@hotmail.com (M.K.); 2 Pediatric Intensive Care, Ege University, İzmir 35100, Turkey; dryazicipinar@gmail.com (P.Y.Ö.); cebecikubramd@gmail.com (K.C.); 3 Pediatric Nephrology, Ege University, İzmir 35100, Turkey; ikaplanbulut@gmail.com

**Keywords:** dialysis, hemolytic uremic syndrome, liver involvement, mortality, pediatric intensive care, plasma exchange

## Abstract

**Highlights:**

**What are the main findings?**

- Liver involvement occurs in 68% of pediatric HUS patients but generally follows a reversible and benign course.
- Patients with liver involvement require ICU admission and plasma exchange more frequently, although it does not independently predict disease severity.

**What are the implications of the main findings?**

- Liver involvement should be interpreted within the broader clinical context and managed supportively.
- AST ≥ 42 U/L may help identify patients at risk for adverse outcomes, guiding clinical monitoring.

**Abstract:**

**Background/Objectives**: Hemolytic uremic syndrome is a thrombotic microangiopathy characterized by hemolytic anemia, thrombocytopenia, and acute kidney injury. Liver involvement in pediatric hemolytic uremic syndrome is increasingly recognized, but its clinical significance remains unclear. This study aimed to evaluate the relationship between liver involvement and clinical outcomes in children with hemolytic uremic syndrome. **Methods**: We retrospectively analyzed 25 pediatric patients diagnosed with hemolytic uremic syndrome. Patients were grouped based on liver involvement, defined by elevated transaminase levels. Demographic, clinical, laboratory, and treatment data were collected. Associations between liver involvement, intensive care unit admission, dialysis, plasma exchange, and length of intensive care unit stay were assessed using statistical analyses. **Results**: Liver involvement was observed in 68% of patients. Although liver involvement was not associated with the need for dialysis, affected patients required plasma exchange and intensive care unit admission significantly more often. An AST cutoff of ≥42 U/L moderately predicted adverse outcomes (AUC: 0.619), while ALT had limited prognostic value (AUC: 0.658). Transaminase levels normalized within a mean of 3.76 ± 1.92 days. No mortality was observed in our cohort, in contrast to previously reported rates of 2–5%. **Conclusions**: Liver involvement in pediatric hemolytic uremic syndrome is common but generally follows a reversible and benign course. While it is associated with increased intensive care unit admission and plasma exchange, it does not independently predict disease severity. These findings emphasize the importance of supportive management and suggest that liver involvement should be interpreted in the context of the overall clinical picture.

## 1. Introduction

Hemolytic uremic syndrome (HUS) is a life-threatening condition predominantly affecting children and is recognized as one of the most common causes of acute kidney injury (AKI) in children [1,2,3]. Classically, HUS is defined by the triad of microangiopathic hemolytic anemia, thrombocytopenia, and acute kidney injury. Although the kidney is the primary organ affected, HUS is increasingly recognized as a multisystem disorder with potential involvement of the central nervous system, pancreas, gastrointestinal tract, and liver [4,5,6].

Liver involvement in HUS, although less frequently studied, has been reported in a substantial proportion of patients. Liver involvement may include elevated transaminases, coagulopathy, cholestasis, and rarely, acute liver failure. These abnormalities are believed to stem from systemic inflammation, endothelial injury, and the formation of microthrombi in hepatic vasculature [7,8]. The pathophysiological basis of hepatic involvement in HUS likely lies in the same thrombotic microangiopathy process that affects the kidneys: endothelial injury, platelet aggregation, and microthrombus formation in the microvasculature. In Shiga toxin–associated HUS (STEC-HUS), Shiga toxin binds to endothelial cells, triggering cytotoxicity and activation of the coagulation cascade [9]. Additionally, complement activation can further damage endothelial cells and promote microthrombi formation in small hepatic vessels [10]. Histopathological reports and case studies support this, showing microthrombi in hepatic sinusoids of patients with HUS [11]. Together, these mechanisms suggest that liver injury in HUS may not be purely passive, but a direct consequence of systemic endothelial dysfunction and complement-mediated thrombotic microangiopathy, similar to other organs. Given that liver involvement may complicate fluid and electrolyte management, influence coagulopathy, and increase the risk of multiorgan failure, understanding its clinical implications is critical. Nevertheless, liver involvement in HUS is underappreciated, and its precise impact on disease severity and clinical outcomes remains insufficiently elucidated. Early recognition of liver involvement could guide risk stratification and help predict the need for intensive interventions such as dialysis, plasma exchange (PEX), or prolonged intensive care unit (ICU) admission. Previous studies have evaluated whether liver transaminases may serve as early prognostic markers in HUS. Talathi et al. [12] reported that AST ≥ 129 U/L and ALT ≥ 83 U/L at admission were independently associated with the need for dialysis in children with STEC (−) HUS. Similarly, other investigators proposed ALT ≥ 70 U/L as a predictor of severe renal involvement [13,14,15]. However, larger cohorts have not consistently confirmed these associations, and in some studies ALT lost significance in multivariate analysis. Therefore, the prognostic value of AST and ALT elevations in HUS remains uncertain.

Despite the potential significance, few studies have systematically examined the prognostic value of hepatic dysfunction in pediatric HUS [8,15,16]. There is a clear gap in the literature regarding the correlation between hepatic parameters and disease progression and outcomes. This study aims to evaluate the frequency and extent of liver involvement in pediatric patients with HUS, as well as its impact on clinical outcomes such as the need for dialysis, plasma exchange, and intensive care unit (ICU) admission.

## 2. Materials and Methods

This retrospective study was conducted at Ege University Faculty of Medicine Children’s Hospital, analyzing pediatric patients (age 0–18 years) diagnosed with HUS. Ethical approval was obtained from the Ege University Ethics Committee prior to data collection (2025-5503). Children diagnosed with HUS based on clinical and laboratory criteria were retrospectively analyzed. Patients with known pre-existing chronic liver disease, other causes of thrombotic microangiopathy, or incomplete medical records were excluded. Patient records were reviewed for demographic characteristics, clinical findings, laboratory results (including liver function tests such as aspartate aminotransferase (AST), alanine aminotransferase (ALT), gamma-glutamyl transferase (GGT), and the international normalized ratio (INR), albumin, total and direct bilirubin), imaging data, treatment modalities, and outcomes.

### 2.1. Definitions

Patients presenting with the classic triad of symptoms—microangiopathic hemolytic anemia, thrombocytopenia, and acute kidney injury—were diagnosed as having HUS. Thrombotic thrombocytopenic purpura is ruled out by measuring ADAMTS-13 levels and activity in all patients diagnosed with HUS. Hemolytic uremic syndrome exhibits a broad etiological distribution and was previously classified as typical and atypical HUS. With a clearer understanding of the pathogenesis of the disease, there has been a consensus in recent years that the classification should be based on the etiological factor causing HUS [17,18]. All patients diagnosed with HUS in our center underwent STEC testing (culture and PCR) as part of our standard protocol. Patients with HUS caused by STEC producing Shiga toxin were grouped as STEC (+) HUS. Patients in whom STEC infection was excluded were grouped as STEC (−) HUS. STEC (−) HUS was defined as cases of HUS that were complement-mediated and/or associated with genetic mutations, or that were non-diarrheal or had secondary causes. Additionally, we perform genetic testing using complement studies (C3 and C4) and a complement gene panel to diagnose complement-mediated HUS in all patients diagnosed with HUS at our center.

Patients were grouped on the presence or absence of liver involvement. Liver involvement was defined as elevation of liver enzymes above 2× the upper limit of normal (ULN). In our institution, the ULN for both AST and ALT is 35 U/L, and this reference range was consistently applied to all patients throughout the study period. For consistency and to avoid potential confounding from drug-related enzyme elevations during hospitalization, transaminase values obtained at the time of admission were used for analysis. Patients with liver involvement were also divided into two groups: those with moderately elevated transaminases (>2–5 times the upper limit of normal) and those with markedly elevated transaminases (>5 times the upper limit).

### 2.2. Statistical Analysis

Prior to statistical analysis, the distribution of continuous variables was assessed using the Shapiro–Wilk test to determine normality. Accordingly, normally distributed variables were presented as mean ± standard deviation (SD), while non-normally distributed variables were expressed as median with interquartile range. Categorical variables were expressed as frequencies and percentages. Comparisons between groups (with and without liver involvement) were performed using the chi-square (χ^2^) test for categorical variables. Depending on data distribution, an independent samples t-test or Mann–Whitney U test was used for continuous variables. Receiver operating characteristic (ROC) curve analysis was conducted to determine optimal cutoff values for ALT and AST levels in predicting the need for dialysis, PEX, and pediatric ICU. Based on the identified thresholds, logistic regression analysis was performed to assess the association between elevated ALT and AST levels and clinical outcomes, including the need for dialysis, PEX, and ICU admission. All statistical analyses were carried out using the Statistical Package for the Social Sciences version 25.0 (IBM Corp., Armonk, NY, USA). A *p*-value of less than 0.05 was considered statistically significant.

## 3. Results

### 3.1. Demographic and Laboratory Data and Imaging of the Patients

A total of 25 children (13 female/12 male) diagnosed with HUS were included in the study. The mean age at diagnosis was 66 ± 52 months. Regarding anthropometric measurements, the mean body weight was 21.6 ± 12.6 kg, with a corresponding mean weight z-score of −0.27 ± 0.97. The mean height was 109.2 ± 27.5 cm, with a mean height z-score of −0.22 ± 0.90. The mean BMI was 16.5 ± 2.5 kg/m^2^.

Among the 25 patients, liver involvement was observed in 17 patients (68%). Eleven (44%) of the patients were classified as STEC (+) HUS, while 14 (56%) were classified as STEC (−) HUS. The majority of patients (n = 19, 76%) required admission to the pediatric ICU, with a median ICU stay of 5.5 days (range: 1–25 days). Dialysis was required in most cases, with hemodialysis performed in 18 patients (72%) and peritoneal dialysis in 3 patients (12%). As for treatment, PEX was administered to 19 patients (76%), eculizumab to 12 patients (48%), and steroids to 2 patients (8%). Clinical and laboratory features of the patients are summarized in Table 1.

Abdominal ultrasonography was performed in 56% of our patients. Among these patients, biliary sludge was detected in two patients, and gallbladder wall edema was observed in one patient.

### 3.2. Impact of Liver Involvement and Its Severity on Patient Characteristics

As shown in Table 2, patients with liver involvement were more likely to require pediatric ICU admission (*p* = 0.037) and undergo PEX (*p* = 0.002) compared to those without liver involvement. Although dialysis requirement was more frequent among patients with liver involvement, this difference was not statistically significant (*p* = 0.400). There was also no significant difference in ICU stay between the groups (*p* = 0.310). Among patients with liver involvement, the median AST was 106 U/L (range: 48–298), and the mean AST was 147.52 ± 93.37 U/L. In contrast, patients without liver involvement had a median AST of 18.5 U/L (range: 11–33) and a mean AST of 26.14 ± 8.66 U/L.

No significant difference was observed between STEC (+) HUS and STEC (−) HUS types in relation to liver involvement (*p* = 0.678). No significant differences were observed between patients with and without liver involvement in terms of urea, creatinine, total bilirubin, direct bilirubin, total protein, albumin, and INR levels.

Among the 17 (68%) patients with liver involvement, the relationship between the degree of transaminase elevation and various clinical features was examined. No significant association was found between HUS type and the severity of liver enzyme elevation (*p* = 0.906), with both STEC (+) HUS and STEC (−) HUS cases observed in each group. Although ICU admission was more frequent among patients with transaminase elevation > 5× (10 patients vs. 5 patients), the difference did not reach statistical significance (*p* = 0.072). Similarly, the dialysis requirement (*p* = 0.787) and PEX (*p* = 0.218) did not differ significantly between the two groups. The median ICU stay was comparable between patients with moderate and marked liver enzyme elevation: 5 days (range: 3–25) vs. 6.5 days (range: 2–24), respectively (*p* = 0.853).

The relationship between ICU stays and ALT and AST levels was assessed. A weak and statistically non-significant positive correlation was found between length of ICU stays and both ALT (r = 0.047, *p* = 0.854) and AST (r = 0.204, *p* = 0.417) levels. The average time for liver enzyme levels to return to normal was 3.76 ± 1.92 days.

Patients were analyzed to assess whether elevated transaminases were associated with clinical severity, particularly the need for PEX, dialysis, and ICU admission. ROC curve analysis was conducted to evaluate the predictive capacity of AST and ALT levels for three major clinical outcomes: PEX requirement, dialysis requirement, and ICU admission in patients with HUS. For AST, a cutoff ≥ 42 U/L showed 75% sensitivity and 75% specificity for predicting PEX, dialysis, and ICU admission, with an area under the curve (AUC) of 0.619. For ALT, a cutoff ≥ 40.5 U/L showed 78.9% sensitivity and 50% specificity for plasmapheresis, and 63.2% sensitivity and 50% specificity for dialysis and ICU admission, with an AUC of 0.658. Both liver enzymes demonstrated moderate discriminatory ability.

Patients were categorized accordingly for logistic regression analysis. In univariate logistic regression analyses, Elevated AST (≥42 U/L) was significantly associated with all three outcomes: PEX requirement (OR: 15.0, *p* = 0.030), dialysis requirement (OR: 15.0, *p* = 0.030), and ICU admission (OR: 15.0, *p* = 0.030). Elevated ALT (≥40.5 U/L) was significantly associated with PEX requirement (OR: 16.0, *p* = 0.035), but not significantly associated with dialysis requirement or ICU admission (OR: 3.75, *p* = 0.249). These findings indicate that AST elevation above 42 U/L is a robust and statistically significant predictor of disease severity in HUS, while ALT elevation above 40.5 U/L shows a significant association only with PEX requirement. These findings are summarized in Table 3.

### 3.3. Mortality

Despite the need for ICU and kidney replacement therapies in many cases, no mortality occurred during the follow-up period.

## 4. Discussion

In this study, we observed that liver involvement was a frequent finding in pediatric HUS, affecting more than two-thirds of patients, yet it appeared to be transient and clinically mild. Importantly, while liver enzyme elevations were not associated with an increased need for dialysis, they showed significant associations with intensive care admission and PEX, suggesting that liver involvement may reflect the systemic severity of the disease rather than isolated hepatic dysfunction. Our analysis further demonstrated that AST elevation above 42 U/L was a strong and consistent predictor of adverse outcomes, whereas ALT elevations showed a more limited prognostic role. Despite the high frequency of severe clinical manifestations requiring ICU support and kidney replacement therapies, no mortality occurred in our cohort, underscoring the potential benefits of early recognition and aggressive supportive care. Together, these findings highlight the relevance of routine liver function monitoring in HUS and provide new insights into the clinical significance of liver involvement in this syndrome.

The pathophysiological mechanisms behind liver involvement in HUS are complex and multifactorial, involving both direct and indirect effects of the underlying disease process. The pathogenesis of HUS begins with endothelial cell injury, which leads to microangiopathic hemolytic anemia and affects not only the renal vasculature but also the hepatic sinusoids, causing liver damage. In cases triggered by STEC, the released toxins directly affect hepatocytes, leading to inflammation and apoptosis. Systemic inflammation, marked by the release of pro-inflammatory cytokines, exacerbates liver dysfunction by impairing hepatocyte function and promoting further endothelial injury. Moreover, the formation of microthrombi within the hepatic vasculature obstructs blood flow, leading to hepatic ischemia and worsening liver injury [1,7,8].

Liver involvement was observed in 68% of patients in our cohort. Giordano et al. [8] analyzed 10 years of STEC-HUS data and reported that hepatic involvement, although less frequent (15 patients, 18.9%), was not uncommon. The lower rate in their study may be explained by their stricter definition of liver involvement as transaminase levels exceeding five times the upper limit of normal, whereas we considered both moderate (2–5× ULN) and marked (>5× ULN) elevations. Notably, they found no correlation between E. coli serotypes or Shiga toxin variants and hepatic involvement, supporting the notion that liver dysfunction in HUS may be influenced more by host factors or overall disease severity than by microbial strain characteristics.

Abdominal ultrasonography was performed in 56% of our patients, detecting biliary sludge in two and gallbladder wall edema in one. In Giordano et al. [8], ultrasonography was performed in 49%, with biliary sludge in three and cholelithiasis in one. In both studies, liver involvement was found to be reversible within approximately one week; in our cohort, transaminase levels returned to normal in 3.76 ± 1.92 days on average. Although hepatic involvement was generally transient and clinically mild in both STEC (+) HUS and STEC (−) HUS, the high frequency of transaminase elevation and associated sonographic findings in our cohort highlight that liver involvement is not rare and may reflect the systemic nature of HUS. These findings reinforce the importance of routine liver function monitoring in HUS patients, even when abnormalities are expected to resolve quickly. 

In our study, patients with elevated liver enzymes were significantly more likely to require plasmapheresis and admission to the ICU. However, hepatic involvement was not associated with an increased need for dialysis. This suggests that while liver involvement may not directly reflect the severity of kidney impairment, it could serve as a marker of a more pronounced systemic inflammatory or immune-mediated process, potentially guiding therapeutic decisions such as the initiation of plasmapheresis. The lack of a significant association between the degree of transaminase elevation and clinical severity indicates that markedly elevated transaminase levels alone may not be a reliable indicator of poor prognosis. Furthermore, the absence of differences between STEC (+) HUS and STEC (−) HUS supports that hepatic involvement is not directly related to HUS subtype. Although transaminase elevations are common in HUS, their prognostic utility remains unclear. Further large-scale, prospective studies are warranted to validate cutoff thresholds and to clarify the clinical significance of liver enzyme levels across different HUS subtypes and patient populations. While our analysis showed that patients with liver involvement were more likely to require ICU admission and plasmapheresis, it is important to acknowledge potential treatment-related confounders. Decisions regarding ICU admission or initiation of PEX were made by the treating physicians based on overall clinical judgment, which may not be entirely captured by laboratory parameters. Therefore, some of the observed associations could partly reflect physician-driven decision-making rather than liver involvement alone. Future prospective studies with standardized treatment protocols could help clarify the independent impact of hepatic involvement on clinical outcomes.

Liver transaminase elevations have been investigated as potential prognostic markers in HUS, but results have been inconsistent across studies. Talathi et al. [12] reported that elevated AST and ALT at admission were independently associated with dialysis requirement in 70 children with STEC (+) HUS, with cutoff values of 129 U/L (AST) and 83 U/L (ALT), showing high specificity. Similarly, another study identified ALT ≥ 70 IU/L as an independent predictor of dialysis [13]. These observations suggest that normal transaminase levels may indicate low risk, whereas markedly elevated values predict the need for kidney replacement therapy, reflecting not only hepatic involvement but also systemic disease severity. However, in a larger cohort of 153 post-diarrheal HUS patients, Balestracci et al. [19] found higher ALT in patients requiring dialysis, but ALT lost significance in multivariate analysis. Kawasaki et al. [14] also proposed an ALT cutoff of 70 IU/L for dialysis prediction in 24 patients.

In our cohort, elevated AST (≥42 U/L; 75% sensitivity and 75% specificity) was significantly associated with plasmapheresis, dialysis, and ICU admission, whereas elevated ALT (≥40.5 U/L; 78.9% sensitivity and 50% specificity) was associated only with plasmapheresis. These findings suggest that AST may be a more reliable early indicator of severe disease in HUS compared to ALT, highlighting the potential value of routine liver function monitoring in these patients.

The mortality rate from HUS has decreased thanks to improved symptomatic treatment. Previous studies have reported a mortality rate of 1–5% among patients with HUS [13,20,21]. In the study conducted by Kamioka et al. [13], only two patients (1.6%) died, one due to acute cardiac failure and the other due to central nervous system complications. In our cohort, no mortality was observed, which may reflect early diagnosis, prompt supportive care, and effective management of complications such as kidney injury and severe systemic involvement. This finding reinforces the generally favorable prognosis of pediatric HUS when managed appropriately, although careful monitoring remains essential.

This study has several limitations. First, the small sample size (n = 25) and single-center retrospective design may limit the statistical power and generalizability of our findings. Second, imaging studies were not completed in all patients, which may have led to an underestimation of hepatic involvement. Third, as a tertiary referral center, our patient population may reflect more severe cases, introducing referral bias. Fourth, standardized diagnostic criteria for STEC (+) HUS versus STEC (−) HUS were not consistently applied across institutions, potentially affecting classification. Finally, the overall generalizability of our results is limited, and larger, multicenter prospective studies are needed to validate our findings.

## 5. Conclusions

Our findings indicate that hepatic involvement is a frequent but generally transient and benign manifestation in pediatric HUS. Although it is associated with a higher need for intensive care and interventions such as plasmapheresis, liver injury itself does not independently predict poorer outcomes. Although mortality from HUS has declined with improved supportive care, recognition of hepatic involvement remains clinically relevant, as it may indicate multisystem involvement and help guide comprehensive patient management. These results underscore the importance of supportive care and suggest that hepatic dysfunction, while common, may not require aggressive liver-specific interventions. Larger prospective studies are warranted to further clarify the prognostic significance and underlying pathophysiology of liver involvement in HUS.

## Figures and Tables

**Table 1:** Clinical and laboratory features of the patients.

Clinical Features	n (%)
Liver involvement (Yes)	17 (68)
HUS Type (STEC HUS/STEC (−) HUS)	11/14 (44/56)
ICU requirement (Yes)	19 (76)
Dialysis requirement	
- Hemodialysis	18 (72)
- Peritoneal dialysis	3 (12)
Treatment	
- PEX	19 (76)
- Eculizumab	12 (48)
- Steroid	2 (8)
**Laboratory features**	**Mean ± SD**
Hemoglobin (g/dL)	8.67 ± 2.11
Urea (mg/dL)	156.72 ± 104.49
Creatinine (mg/dL)	3.81 ± 3.35
AST (U/L)	112.12 ± 96.28
ALP (U/L)	122.83 ± 50.84
Total protein (g/dL)	5.36 ± 1.00
	**Median (min–max)**
White blood cell (×10^3^/μL)	10.32 (4.64–57.09)
Platelet (×10^3^/μL)	59.0 (4–78.1)
ALT (U/L)	90 (11–298)
Total bilirubin (mg/dL)	0.7 (0.15–3.97)
Direct bilirubin (mg/dL)	0.22 (0.05–1.02)
GGT (U/L)	10 (5–264)
Albumin (g/dL)	3 (2.40–11.80)
INR	1.02 (0.90–1.65)
**ICU stay (days)**	5.5 (1–25)

ALP: Alkaline phosphatase, ALT: Alanine aminotransferase, AST: Aspartate aminotransferase, GGT: Gamma-glutamyl transferase, HUS: Hemolytic uremic syndrome, INR: International normalized ratio, ICU: Intensive Care Unit, PEX: plasma exchange.

**Table 2:** Clinical Comparison Based on Liver Involvement and Transaminase Elevation Severity.

Patients (n = 25)	Liver Involvement (+) (n = 17)	Liver Involvement (−) (n = 8)	*p* Value	Transaminase Level >2×–5× (ULN) (n = 7)	Transaminase Level >5× (ULN) (n = 10)	*p* Value
**HUS Type** (STEC (+) HUS/STEC (−) HUS)	7/10	4/4		3/4	4/6	0.906 *
**ICU Requirement** **(Yes)**	15	4	0.037 *	5	10	0.072 *
**Dialysis Requirement (Yes)**	15	6	0.400 *	6	9	0.787 *
**PEX Requirement (Yes)**	16	3	0.002 *	6	10	0.218 *
**ICU stay (days)** **Median (min**–**max)**	6 (2–25)	3 (1–7)	0.310 ^#^	5 (3–25)	6.50 (2–24)	0.853 ^#^

* Chi-square test, ^#^ Mann–Whitney U test; HUS: Hemolytic Uremic Syndrome, ICU: Intensive Care Unit, PEX: plasma exchange, ULN: Upper Limit of Normal.

**Table 3:** Association of AST and ALT with Clinical Outcomes.

Predictor	Outcome	OR/*p*-Value
***AST ≥ 42 U/L***	PEX Requirement	15.0/***0.030***
***AST ≥ 42 U/L***	Dialysis Requirement	15.0/***0.030***
***AST ≥ 42 U/L***	ICU Admission	15.0/***0.030***
***ALT ≥ 40.5 U/L***	PEX Requirement	16.0/***0.035***
***ALT ≥ 40.5 U/L***	Dialysis Requirement	3.75/0.249
***ALT ≥ 40.5 U/L***	ICU Admission	3.75/0.249

AST: aspartate aminotransferase, ALT: alanine aminotransferase, ICU: intensive care unit, PEX: plasma exchange, OR: odds ratio. Logistic regression (univariate) was used for analysis.

## Data Availability

The original contributions presented in this study are included in the article. Further inquiries can be directed to the corresponding author.

## Abbreviations

The following abbreviations are used in this manuscript:

HUS	Hemolytic Uremic Syndrome
AKI	Acute Kidney Injury
PEX	Plasma Exchange
ICU	Intensive Care Unit
AST	Aspartate Aminotransferase
ALT	Alanine Aminotransferase
GGT	Gamma-Glutamyl Transferase
INR	International Normalized Ratio
ULN	Upper Limit of Normal
